# Salt Tolerant and Sensitive Rice Varieties Display Differential Methylome Flexibility under Salt Stress

**DOI:** 10.1371/journal.pone.0124060

**Published:** 2015-05-01

**Authors:** Liliana J. Ferreira, Vanessa Azevedo, João Maroco, M. Margarida Oliveira, Ana Paula Santos

**Affiliations:** 1 Instituto de Tecnologia Química e Biológica António Xavier, Universidade Nova de Lisboa, Genomics of Plant Stress. Av. da República, 2780–157 Oeiras, Portugal; 2 UIPES, ISPA-Instituto Universitário, Lisbon, Portugal; Louisiana State University Agricultural Center, UNITED STATES

## Abstract

DNA methylation has been referred as an important player in plant genomic responses to environmental stresses but correlations between the methylome plasticity and specific traits of interest are still far from being understood. In this study, we inspected global DNA methylation levels in salt tolerant and sensitive rice varieties upon salt stress imposition. Global DNA methylation was quantified using the 5-methylcytosine (5mC) antibody and an ELISA-based technique, which is an affordable and quite pioneer assay in plants, and *in situ* imaging of methylation sites in interphase nuclei of tissue sections. Variations of global DNA methylation levels in response to salt stress were tissue- and genotype-dependent. We show a connection between a higher ability of DNA methylation adjustment levels and salt stress tolerance. The salt-tolerant rice variety Pokkali was remarkable in its ability to quickly relax DNA methylation in response to salt stress. In spite of the same tendency for reduction of global methylation under salinity, in the salt-sensitive rice variety IR29 such reduction was not statistically supported. In ‘Pokkali’, the salt stress-induced demethylation may be linked to active demethylation due to increased expression of DNA demethylases under salt stress. In ‘IR29’, the induction of both DNA demethylases and methyltransferases may explain the lower plasticity of DNA methylation. We further show that mutations for epigenetic regulators affected specific phenotypic parameters related to salinity tolerance, such as the root length and biomass. This work emphasizes the role of differential methylome flexibility between salt tolerant and salt sensitive rice varieties as an important player in salt stress tolerance, reinforcing the need to better understand the connection between epigenetic networks and plant responses to environmental stresses.

## Introduction

Soil salinity is a major environmental constraint to crop production with negative impacts on growth rates, tillering and seed production [1]. Rice (*Oryza sativa L*.) is the world's most important food crop facing particular problems with cultivation under adverse climate conditions. Due to the large genetic variability, rice varieties possess different degrees of salt sensitivity [2]. Salinity tolerance is a quantitative trait controlled by multiple genes [3] which can be involved in signal transduction, ion transportation, metabolic pathways and transcription regulation [4]. Epigenetic mechanisms such as DNA methylation, histone modifications, nucleosome positioning and small non-coding RNAs, act in coordination to influence chromatin structure and gene expression [5–8]. DNA methylation levels are modulated by an intricate interplay of DNA methyltransferases, DNA demethylases and other mechanisms, such as RNA-directed DNA methylation (RdDM) pathway, mediated by siRNAs but little is known regarding the mechanistic process of establishing, maintaining and removing methylation marks [9]. Also, the functional significance of DNA methylation in plant response to environmental stress conditions is still largely unknown. Variations of epigenetic patterns and chromatin structure in relation to environmental conditions have been inspected by molecular and cytological approaches. Fluorescence *In Situ* Hybridization (FISH) enabled to detect major reorganization of highly condensed heterochromatic domains as ribosomal chromatin after imposing salt stress or hypomethylating agents e.g. 5-azacytidine (5-AC) [10]. The methylation-sensitive amplified polymorphism (MSAP) technique has been used to study the impact of stress on global DNA methylation [11–14].

This work focused in evaluating global DNA methylation levels in rice varieties with contrasting behaviors in response to salt stress. Shifts on global DNA methylation were detected after salt stress imposition. In addition, these shifts were influenced by genotype and tissue specificity. While the salt tolerant rice variety Pokkali was able to rapidly reduce DNA methylation under salt stress, the salt sensitive variety IR29 did not show such methylome flexibility, suggesting a link between the plasticity of DNA methylation and plant performance under salt stress. Furthermore, mutations of epigenetic modulators affected specific phenotypic parameters related to salinity tolerance such as root length and biomass.

## Materials and Methods

### Plant material, growth conditions and salt stress treatments

Salt tolerant and sensitive rice varieties (*Oryza sativa* L.) were provided by the International Rice Gene Bank held at the International Rice Research Institute (IRRI), Philippines. The ‘IR29’ is an *indica* variety referred as salt-sensitive standard [15]. ‘Pokkali’ is also an *indica* variety and has been widely used as salt-tolerant donor in breeding programs [16]. The ‘FL478’, also known as IR66946-3R-178-1-1, is a salt tolerant recombinant inbred line developed at IRRI and was used because their parents are ‘Pokkali’ and ‘IR29’ [17]. The *japonica* variety Nipponbare has been described as salt susceptible [13].

Rice mutants for epigenetic regulators were originally developed by Gynheung An (Kyung Hee University, Crop Biotech Institute, Korea). In this study, the T-DNA insertion lines, 4A-01884 and 3A-08043 carrying mutations for a histone acetyltransferase and a DNA methyltransferase, respectively were used [18,19]. The Dongjin variety described as relatively salt tolerant [20] was also used since it is the wild-type from which these mutants originated.

Rice seeds were surface sterilized in 0.1% Benlate solution for 30 min at 50°C, rinsed with sterile water, soaked in 70% ethanol for 1 min and washed with a solution of 2% sodium hypochlorite containing 0.02% Tween 20 for 30 min. After successive washings in sterile water, the seeds were placed on filter paper soaked in water and allowed to germinate for 3 days at 28°C. Germinated seedlings were transferred to a hydroponic system containing Yoshida’s medium [21]. Plants were allowed to grow in a growth-chamber at 28°C/24°C in a 12h photoperiod regime (500 μEm^-2^s^-1^) and with 70% humidity. The salt stress was imposed on 14-days-old rice seedlings by supplementing Yoshida’s medium with 200 mM NaCl (EC = 24 dS/m; EC = electrical conductivity). Root and leaf tissues were collected separately after 1 and 24h of salt treatment, frozen in liquid nitrogen and kept at -80°C.

Seeds from rice mutants were heat-treated for 5 days in a convection oven set at 50°C to break seed dormancy, then placed on filter paper soaked in water and incubated at 28°C in the dark for 48h to germinate. The experimental design was a split plot design with 5 lines per tray and 26 plants per genotype. Two pre-germinated seeds were sown per hole on a styrofoam seedling float device and the emerging radicle was carefully inserted through the nylon mesh. The styrofoam device was suspended on a tray filled with only distilled water, since the endosperm has enough nutrients for the seedlings to grow normally for 3–4 days. After this period, trays were filled with Yoshida solution [21]. Two distinct salinity assays were performed. In “Assay 1”, 4-days-old seedlings were subjected to an initial salinization of EC = 6 dS/m during one week that was then increased to EC = 12 dS/m for another week. In “Assay 2”, the salt imposition was applied in 12-days-old seedlings, with an initial salinization of EC = 6 dS/m for 3 days followed by a week in EC = 12 dS/m. In the control trays no salt was added. The solutions were renewed every 8 days and the pH daily checked and maintained at 5.0. The experiment was conducted in the growth chamber as mentioned above. All trays had two check entries: the susceptible ‘IR29’ and the tolerant ‘Pokkali’.

### Quantification of global DNA methylation

A relative quantification of global DNA methylation levels was obtained with an ELISA-based colorimetric assay using a commercially available kit, the MDQ1 (Imprint Methylated DNA Quantification kit, Sigma Aldrich). The procedure was according to manufacturer's instructions. Even though some differences in methylation can occur between replicates, the use of multiple biological and technical replicates allowed a consistent calculation of the relative amounts of DNA methylation. Genomic DNA of roots and leaves from rice lines was isolated by using the DNeasy Plant mini kit (Qiagen). Three independent DNA extractions were performed from a pool of twelve 14-days-old rice seedlings subjected to 1 or 24h of salt treatment. One hundred nanograms of genomic DNA per sample was immobilized on strip wells with high affinity for DNA and incubated at 60°C. The methylated DNA was detected using optimized antibody and reagents with high specificity to 5mC and then quantified colorimetrically. The absorbance was read on a microplate reader at 450 nm (Biotek Power Wave XS). The percentage of DNA methylation was calculated relative to an internal standard methylated control DNA supplied in the kit and according to the manufacturer’s protocol. The following steps were followed: (a) average the A_450_ replicates for the blank, samples and methylated control DNA and (b) use the formula [(A_450 av_ sample—A_450 av_ blank)/(A_450 av_ methylated control DNA—A_450 av_ blank)] x 100 for calculation of % methylation of the samples relative to the methylated control DNA.

### Imaging of 5-methylcytosine in interphase nuclei of tissue sections

Root-tips of 14-days-old seedlings were excised and fixed in 4% (w/v) formaldehyde freshly prepared from paraformaldehyde in PEM buffer (50 mM PIPES; 5 mM EGTA; 5 mM MgSO4; KOH pH 6.9) for 1h at room temperature and then washed in TBS for 10 min. Root tips were sectioned using a Vibratome Series 1000 (TAAB Laboratories Equipment Ltd, Aldermarston, UK) and allowed to dry on multi-well slides (Menzel-Glaser). Root sections of approximately 15–20 μm thick containing about two cell layers showed good tissue preservation and integrity. The slides were pre-treated by washing in 3% Decon90 (detergent) for at least 1h, thoroughly rinsed with distilled water and coated with a freshly prepared solution of 2% (v/v) 3-aminopropyltriethoxysilane (APTES, Sigma) in acetone for 10 s and activated with 2.5% (v/v) glutaraldehyde (Sigma) in PBS for 30 min, rinsed in distilled water and air-dried. Prior to immunofluorescence, tissue sections were dehydrated in an ethanol series and digested with an enzyme mixture of cellulase 1.5% (w/v) (Onozuka R-10, Japan) and pectolyase 0.5% (w/v) (Sigma) in EB (0.4 mM citric acid; 0.6 mM trisodium citrate, pH 4.8) for 1h at room temperature. After washing 10 min with PBS, the sections were permeabilized with 0.1% Triton X-100 (v/v) in PBS for 3 min, washed again 10 min in PBS and dehydrated in an ethanol series and air-dried.

For *in situ* detection of 5mC, a mouse monoclonal antibody against 5-methylcytosine (1:100, Santa Cruz Biotechnology) was used, followed by a secondary antibody (goat anti-mouse Alexa Fluor 488 1:500, Invitrogen). After blocking with 1% (w/v) Bovine Serum Albumine (Roche) dissolved in PBS/0.1% Triton X-100 for 1h, the tissue sections were incubated with the primary antibody overnight at 4°C. After washes in PBS/0.1% Triton X-100, incubation with the secondary antibody was performed at 37°C for 90 min. The nuclei were counterstained with DRAQ5 (1:1000 in PBS, Cell Signaling Technology) for 10 min, rinsed briefly in PBS and mounted in Vectashield antifade solution (Vector Laboratories). Confocal optical section stacks (z-step size of 1 μm) were collected with a Leica TCS SP confocal microscope (Leica Microsystems, Heidelberg GmbH). The microscopy data were transferred to Image J and processed using constant parameters to minimize operator errors. The fluorescence intensity was measured in Z-projections obtained by using the maximum intensity of each stack individually. At least 65 nuclei were measured for each condition. The arbitrary units correspond to the values of the Raw Integrated Density measurement. Finally, Adobe Photoshop 5.0 (Adobe systems Inc., Mountain View, CA) was used for image composition.

### Expression studies of DNA methyltransferases and demethylases by real-time quantitative PCR (qPCR)

Total RNA from roots and leaves of ‘Pokkali’ and ‘IR29’ was isolated from a pool of twelve 14-days-old rice seedlings subjected to either 1 or 24h of salt imposition (200 mM NaCl). The RNA extraction procedure followed the manufacturer’s instructions (Zymo Resarch). The isolated total RNA was treated with TURBO DNA-free (Ambion) to eliminate any possible DNA trace. The cDNA first strand was synthesized from 4 μg of total RNA using the Randon Hexamer primer and according to the instructions from the Transcriptor High Fidelity cDNA Synthesis Kit (Roche). Real-time quantitative PCR was performed using the LightCycler 480 system (Roche) and the SYBR Green I Master mix (Roche). The PCR running conditions were as follows: one cycle at 95°C for 5 min and 45 cycles of amplification at 95°C for 10 s, 60°C for 10 s and 72°C for 10 s. All qPCR experiments were performed on two biological replicates and the CT values were calculated from means of three technical replicates. The relative quantification of gene expression was calculated with kinetic PCR efficiency correction using the comparative Ct method (2(-ΔΔCt) to determine the relative expression of transcripts relative to an endogenous control. The rice gene Ubiquitin-conjugating Enzyme E2 (*OsUBC2*, LOC_Os02g42314) was used as endogenous gene, due to its stability under salt stress, to normalize the relative expression of the target transcripts (S4 Fig). The qPCR reactions were performed with specific primers for the DNA demethylases DNG701 and DNG710, and for the DNA methyltransferase OsDRM2, listed on S1 Table.

### DNA methylation analysis by McrBC digestion

Genomic DNA from ‘Pokkali’ and ‘IR29’ leaves was isolated as referred above. Genomic DNA (1.5 μg) was digested overnight at 37°C with 25 units of McrBC enzyme (New England Biolabs) in a final volume of 50μl following the manufacturer’s instructions. Digested and negative control samples were subjected to PCR amplification to detect the methylation status of eight selected transposable elements (TE-I_Os04g19320, TE-I_Os04g17620, TE-II_Os04g087100, Chr3-AnacA2_TE, Chr8-Tnr8_TE, Chr9-Ty3/gypsy_TE, Chr12-centromeric-like_LTR, Tos17), one repetitive sequence (Telomere_repetitive seq), four selected salt-stress responsive genes (*OsRMC*, *OsHKT5; OsSalT*, *OsNHX1*) and two constitutively expressed genes (*eEF*, *OsActin*). The primers were designed with NCBI software (http://www.ncbi.nlm.nih.gov/tools/primer-blast/). The sequences are indicated in the S2 Table.

### Genotyping rice T-DNA insertion lines

T-DNA insertion lines (4A-01884 and 3A-08043) carrying mutations for a histone acetyltransferase and a DNA methyltransferase, respectively, were PCR-genotyped to confirm the T-DNA insertion, using the flanking primer sets indicated in S3 Table. According to the supplier, in the line 3A-08043, the insertion site is located in the sixth exon of *OsDRM2* (LOC_Os03g02010) and in the line 4A-01884, in the fourth exon of *OsHAC704* (LOC_Os06g49130) (S1 Fig). Total RNA extracted from the homozygous plants was used to confirm the silencing of *OsDRM2* (a DNA methyltransferase) and *OsHAC704* (a histone acetyltransferase) genes through semi-quantitative RT-PCR (S2 Fig).

### Phenotypic evaluation of rice plants with mutations for epigenetic regulators

At panicle emergence, at least 10 randomly selected panicles were harvested and the number of filled and empty grains was recorded. Spikelet fertility were estimated as the ratio of number of filled grains to total number of reproductive sites (florets) and expressed as percentage. The progeny of homozygous mutant plants was evaluated for salinity tolerance at the seedling stage as previously described. The Modified Standard Evaluation System (SES) for rice was used to rate the visual symptoms of salt injury as described by [22]. The shoot length (cm) was measured from the base of the stem to the tip of the topmost leaf of the plants. Root length (cm) was measured for each plant. Fresh weight (FW) of shoots and roots were determined immediately after collecting the samples, followed by tissue drying at 50°C for 7 days for the determination of dry weight (DW). Water content was estimated as follows %WC = (FW-DW)/DW)*100 and biomass refers to the dry weight. All parameters are presented as percentage relative to control plants.

### Statistical data analysis

All results were statistically analyzed with one-way ANOVA after checking for assumptions (normality with the Shapiro-Wilk test and Homoscedasticity with the Leven test). Where the ANOVA revealed statistically significant effects, the Tukey-HSD test was also used to identify the groups where the values obtained differed. All statistical analysis was conducted with SPSS Statistics (v. 21, SPSS An IBM Company, Chicago, IL). Statistical significance was assumed for p<0.05.

## Results

### Salt stress induced DNA demethylation

**Fig 1:**
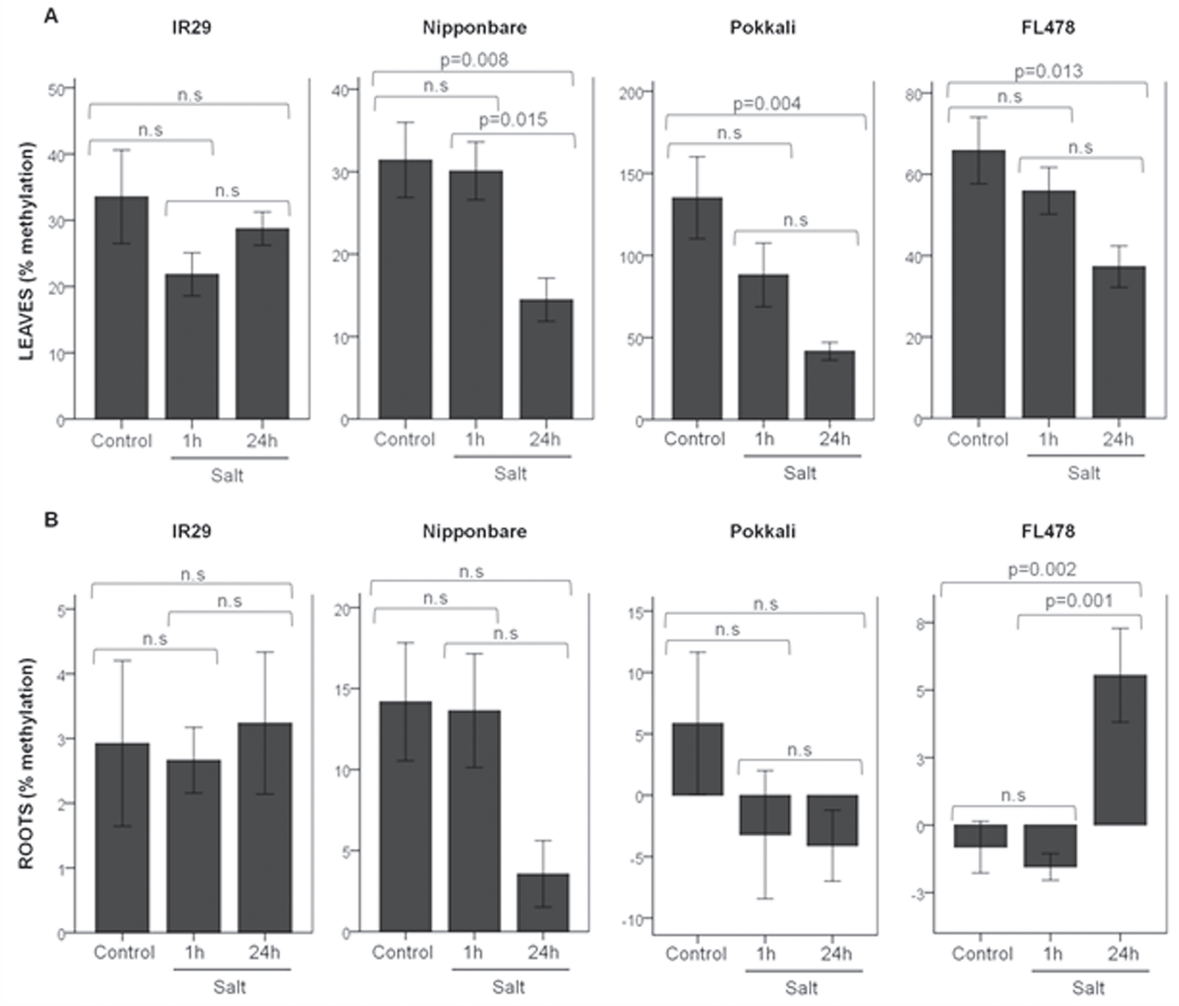
Global DNA methylation levels in salt tolerant and sensitive rice varieties. Genomic DNA from leaves and roots of ‘IR29’, ‘Nipponbare’, ‘Pokkali’ and ‘FL478’ in control and salt stress conditions (1h and 24h of 200 mM NaCl) were used to determine the relative global DNA methylation using a commercial ELISA-based kit. The methylation values represented in the plots correspond to percent methylation of the samples relative to a methylated control DNA supplied with the kit. (A) In leaves, there were statistically significant differences between control and 24h of salt stress in the Nipponbare, Pokkali and FL478 varieties (F(2,24) = 6.68: p = 0.005; F(2,24) = 6.39: p = 0.006; F(2,24) = 4.993: p = 0.015, respectively). (B) For the methylation % in the roots of Nipponbare at 24h of salt stress imposition, there was a significant effect of the treatment on the roots percentage methylation (F(2,24) = 3.601; p = 0.043), although the Tukey’s HSD test only revealed marginally significant differences between the control and 24h treatments (p = 0.06). In ‘FL478’ the methylation % was higher in 24h of salt stress than in control (F(2,24) = 10.946; p<0.001). Statistical significance was assumed for p<0.05. The “p” value was calculated according to the Tukey HSD. N.S: Not Statistically Significant.

The quantification of global amount of 5mC was performed in root and leaf tissues of distinct rice varieties under salt stress to assess tissue and genotype specificity. A common observation to all rice varieties analyzed is the differential methylation levels between roots and leaves. Global DNA methylation levels are lower in roots than in leaf tissues (Fig 1A and 1B).

In leaves, a short exposure to salt stress (1h) was sufficient to cause a decrease of DNA methylation, although not statistically significant which continued to decline upon a more extended stress imposition (24h) except for ‘IR29’ (Fig 1A). The salt tolerant ‘Pokkali’ showed a remarkable ability to alter DNA methylation levels with a 70% decline of total DNA methylation upon salt stress. In ‘Nipponbare’ and ‘FL478’ the methylation loss was about 54% and 43%, respectively. In contrast, in the salt susceptible ‘IR29’, the methylation loss under salinity was only 14%, with no-statistical significance (Fig 1A).

In roots, the effect of salt stress on global DNA methylation was not such conspicuous, with exception for ‘FL478’, which showed statistically significant changes in global DNA methylation in response to 24h of salt stress imposition (Fig 1B).

**Fig 2:**
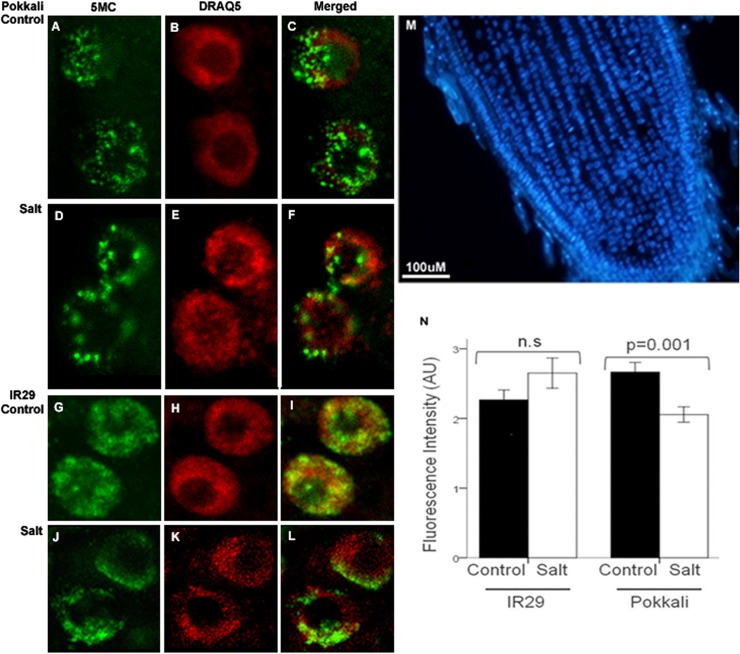
3D imaging of DNA methylation in single interphase nuclei. Immunofluorescence with a specific antibody against 5-methylcytosine was performed in rice root sections of ‘Pokkali’ (A-F) and ‘IR29’ (G-L) 14-days-old seedlings in control conditions or after 24h of exposure to salinity stress (200 mM NaCl). Bar = 10 μM. (M) Rice root-tip section obtained with a vibratome, and stained with DAPI. (N) Fluorescence intensity was calculated using Image J. The values obtained are shown as arbitrary units (AU).

The visualization of DNA methylation loci at single interphase nuclei of root tissues though immunofluorescence with a specific antibody against 5mC was achieved in root sections of ‘Pokkali’ and ‘IR29’ with 14-days-old seedlings in control and stress conditions (24h NaCl). The 5mC loci distribution pattern in rice interphase nuclei consisted of widespread bright fluorescent spots indicating methylation (Fig 2A–2L). An important observation is that salt stress triggered a reorganization of spatial patterns of methylation loci. In addition, the fluorescence intensity was measured and is presented in Arbitrary Units (AU). There was a statistically significant interaction between variety and salt treatment (F(1,391) = 11.095; p = 0.001), meaning that the varieties responded differentially to salt stress (Fig 2N). However, after analyzing the interaction effects only ‘Pokkali’ showed a statistically significant decrease of visible methylation loci in salt stress (F(1, 208) = 12.213; p = 0.001) (Fig 2N).

### Salt-stress effects on DNA methyltransferases and demethylases expression patterns were genotype specific

To better understand DNA methylation dynamics under salt stress, the expression patterns of a rice DNA methyltransferase (MTase) and two DNA demethylases were studied in leaf and root tissues from ‘Pokkali’ and ‘IR29’ in control conditions or after salt stress imposition. The OsDRM2 is a DNA methyltransferase required for *de novo* methylation (encoded by LOC_Os03g02010), while the DNG701 (encoded by LOC_Os05g37350) and DNG710 (encoded by LOC_Os05g50290) act as putative DNA glycosylases/lyases involved in targeted removal of 5mC from methylated DNA [23,24].

**Fig 3:**
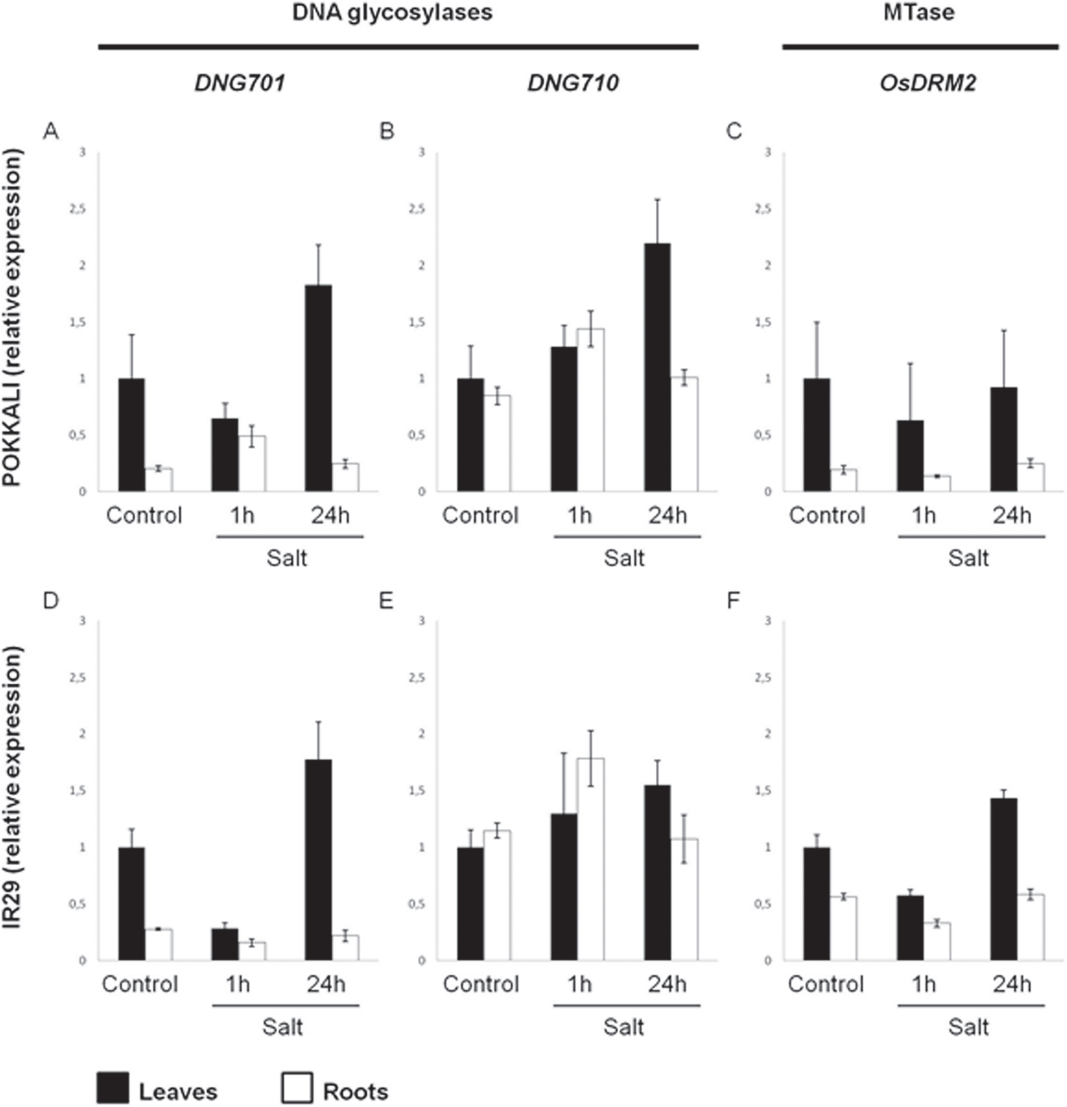
DNA demethylases (*DNG701* and *DNG710*) and DNA methyltransferase (*OsDRM2*) expression studies. Quantitative real-time PCR reactions were performed with cDNA prepared from total RNA extracted from leaves and roots of 14-days-old seedlings of ‘Pokkali’ (A-C) and ‘IR29’ (D-F) subjected to salinity for 1h or 24h (200 mM NaCl). The data were normalized to the internal control Ubiquitin-conjugating Enzyme E2 (*OsUBC2)*. The mean expression value of control was normalized to 1 and the other means values represent fold change in expression. Error bars represent standard deviation.

In leaves, the *DNG701* expression pattern was basically similar in both varieties with a decrease after 1h of salt stress and an increase after a more prolonged exposure to salt (24h) (Fig 3A and 3D). Regarding the *DNG710*, a gradual increase in expression was detected in leaves of both varieties along salt stress imposition (Fig 3B and 3E). Concerning the methyltransferase (*OsDRM2*), the most striking observation is the distinct expression profiles of ‘Pokkali’ and ‘IR29’ under prolonged exposure to salt. The *OsDRM2* expression level is increased in ‘IR29’ but not in ‘Pokkali’ (Fig 3C and 3F). For ‘IR29’, a statistically significant interaction exists between treatment and gene expression (F(4,18) = 41.3; p<0.001), as well as between treatments (control and the two stress periods) (F(2,18) = 56.601; p<0.001). Similarly, in ‘Pokkali’ a statistically significant interaction was found between gene expression and treatment (F(4,18) = 10.647; p<0.001) or between treatments (F(2,18) = 23.085; p<0.001).

In what concerns to root tissues, a differential expression profile of demethylases was detected between the two varieties under analysis. In ‘Pokkali’, salt stress induced the expression of demethylases (Fig 3A and 3B) while in ‘IR29’ the *DNG701* showed declining expression (Fig 3D). Regarding the methyltransferase, a significant repression was detected after 1h of salt stress (Fig 3C and 3F). A more prolonged exposure to salt (24h) was enough to recover the expression of demethylases and methyltransferase to the original levels. Statistical data analysis revealed that ‘IR29’ showed a significant interaction between gene and treatment (F(4,18) = 13.367; p<0.001) but the differences between treatments were not significant (F(2,18) = 0.739; p = 0.492). ‘Pokkali’ showed a statistically significant interaction between genes and treatment (F(2,18) = 8.397; p = 0.001), as well as between treatments (F(2,18) = 8.397; p<0.001).

### DNA methylation of stress related targets

**Fig 4:**
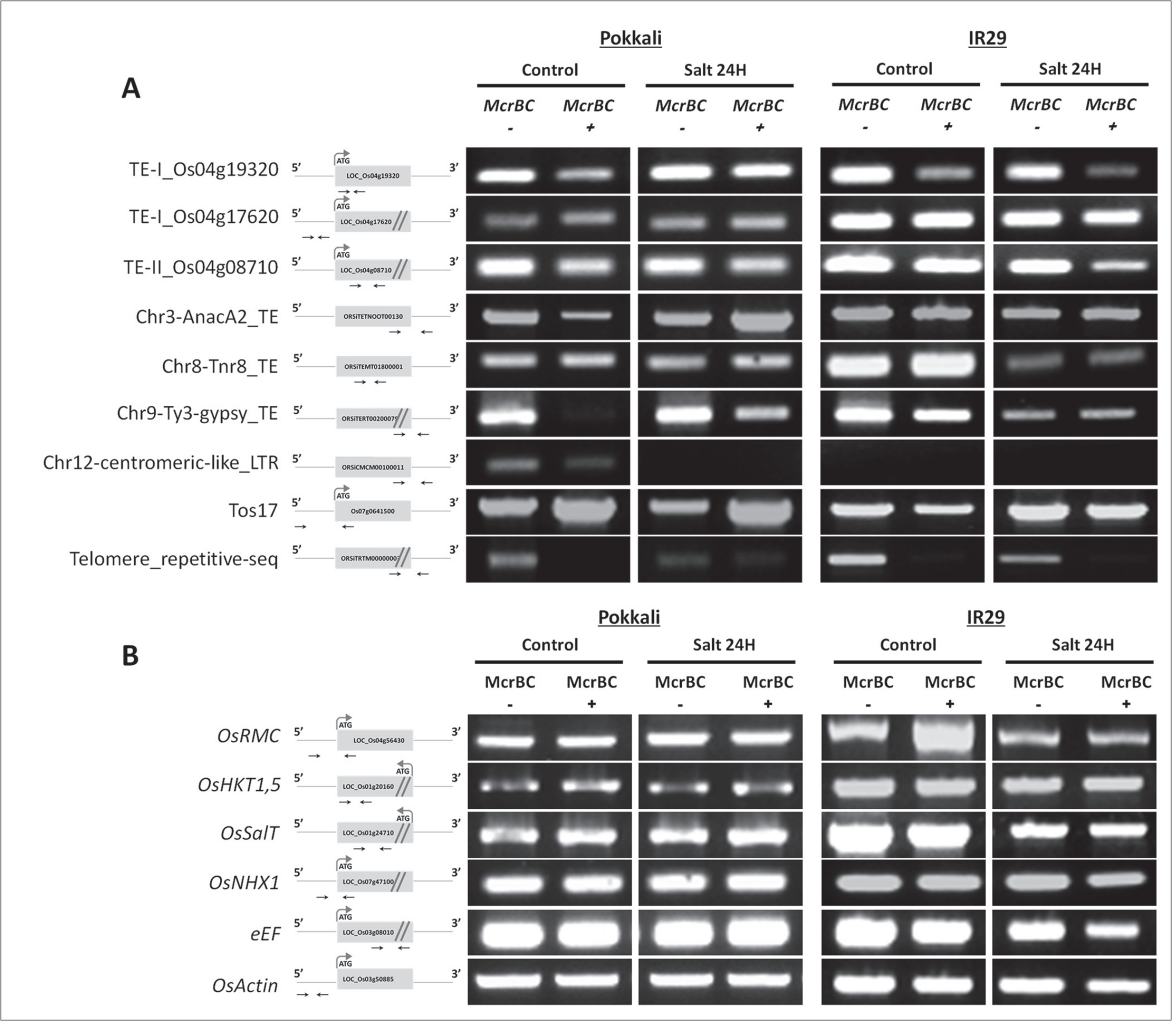
McrBC based methylation analysis. Leaves of Pokkali and IR29 rice varieties in control and salt stress conditions (24h of 200 mM NaCl) were used. (A) Transposable elements (TEs) and repetitive sequences (B) Genes involved in rice responses to salt stress and constitutively expressed genes. The position of primers for all selected TE elements and stress related genes is indicated.

To evaluate whether salt stress-induced demethylation was preferentially occurring at transposable elements (TEs), repetitive sequences or specific salt-stress related genes, a McrBC digestion followed by methylation-sensitive PCR (MS-PCR) was performed in Pokkali and IR29 varieties. The McrBC is an endonuclease that binds the methylated half-sites (G/A)^m^C and cleaves between them [25]. Successful amplification after digestion indicates lack of methylation. In control conditions, McrBC digestion detected methylation of a Ty3-gypsy TE located on chromosome 9 of “Pokkali” only and a telomeric repetitive sequence in ‘Pokkali’ and ‘IR29’ (Fig 4A). While in ‘Pokkali’ both sequences suffered demethylation under salt stress, in ‘IR29’ the methylation status of the telomeric repetitive sequence was not altered by salt stress (Fig 4A). In addition, the LTR sequence located on Chr12 failed to amplify in control and stress conditions in ‘IR29’ while in ‘Pokkali’ that occurred only under salt stress conditions. Regarding specific salt-stress related genes, the McrBC digestion did not indicate methylation in control or salt stress conditions (Fig 4B).

### Mutations of epigenetic modulators affected phenotypic parameters related to salinity tolerance

To investigate a putative connection between chromatin remodelling enzymes and phenotype under salt stress, specific T-DNA insertion lines were submitted to genotyping. The following homozygous lines were selected, one encoding a histone acetyltransferase (*oshac704*) and other encoding a DNA methyltransferase (*osdrm2*). The *osdrm2* mutant plants (but not the *oshac704*) showed impaired seed germination (roughly only 50% of the seeds were viable, data not shown). Additionally, the *osdrm2* seeds required more than 3 days after imbibition for coleoptile emergence while the WT (Dongjin) seeds only required 36 to 48h. The negative effect in seed germination affected subsequent growth, particularly root length and biomass (Table 1). In contrast, the differences between *oshac704* and WT plants were mostly observed at the shoot level, with mutant plants showing a higher shoot development (Table 1). Both *oshac704* and *osdrm2* mutants had reduced spikelet fertility about half of that observed for WT plants grown under normal conditions as shown in S3 Fig.

**Table 1:** Phenotypic evaluation of rice mutants.

	"Assay 1"						"Assay 2"					
	Control			Salt stress			Control			Salt stress		
	WT	*osdrm2*	*oshac704*	WT	*osdrm2*	*oshac704*	WT	*osdrm2*	*oshac704*	WT	*osdrm2*	*oshac704*
**S.E.S**	2	2	2	6	8	7	1	2	2	6	5	5
**Shoot length (cm)**	35.1 ± 1.74	32.94 ± 5.70	39.7 ± 2.58	19.35 ± 2.37	16.10 ± 2.33	16.00 ± 1.76	43.35 ± 2.48	41.70 ± 6.03	45.61 ± 4.08	35.40 ± 2.30	32.59 ± 2.54	35.70 ± 4.23
**Shoot FW (g)**	0.69 ± 0.05	0.62 ± 0.12	0.76 ± 0.13	0.14 ± 0.02	0.09 ± 0.02	0.10 ± 0.02	0.76 ± 0.05	0.48 ± 0.11	0.68 ± 0.07	0.32 ± 0.03	0.20 ± 0.03	0.34 ± 0.05
**Shoot DW (g)**	0.11 ± 0.02	0.11 ± 0.05	0.11 ± 0.02	0.04 ± 0.003	0.03 ± 0.004	0.03 ± 0.003	0.10 ± 0.01	0.06 ± 0.02	0.09 ± 0.02	0.05 ± 0.005	0.05 ± 0.01	0.05 ± 0.01
**Shoot Water Content (%)**	84.34	81.77	85.84	73.99	72.45	74.63	86.84	87.02	86.69	84.93	83.05	85.46
**Root length (cm)**	13.55 ± 0.06	12.50 ± 1.27	12.95 ± 0.90	13.25 ± 2.10	12.45 ± 1.48	8.05 ± 1.55	17. 03 ± 1.52	14.87 ± 2.50	16.08 ± 1.97	11.93 ± 0.91	11.28 ± 1.71	11.60 ± 1.61
**Roots FW (g)**	0.38 ± 0.033	0.31 ± 0.055	0.36 ± 0.065	0.103 ± 0.005	0.085 ± 0.015	0.075 ± 0.012	0.43 ± 0.03	0.28 ± 0.07	0.32 ± 0.06	0.23 ± 0.03	0.17 ± 0.01	0.20 ± 0.04
**Root DW (g)**	0.035 ± 0.003	0.023 ± 0.02	0.039 ± 0.01	0.01 ± 0.0004	0.01 ± 0.002	0.01 ± 0.004	0.06 ± 0.05	0.02 ± 0.005	0.03 ± 0.01	0.02 ± 0.002	0.02 ± 0.001	0.02 ± 0.004
**Root Water Content (%)**	90.81	92.64	89.11	86.55	87.04	84.86	85.48	91.64	90.88	90.11	90.18	90.11

**Fig 5:**
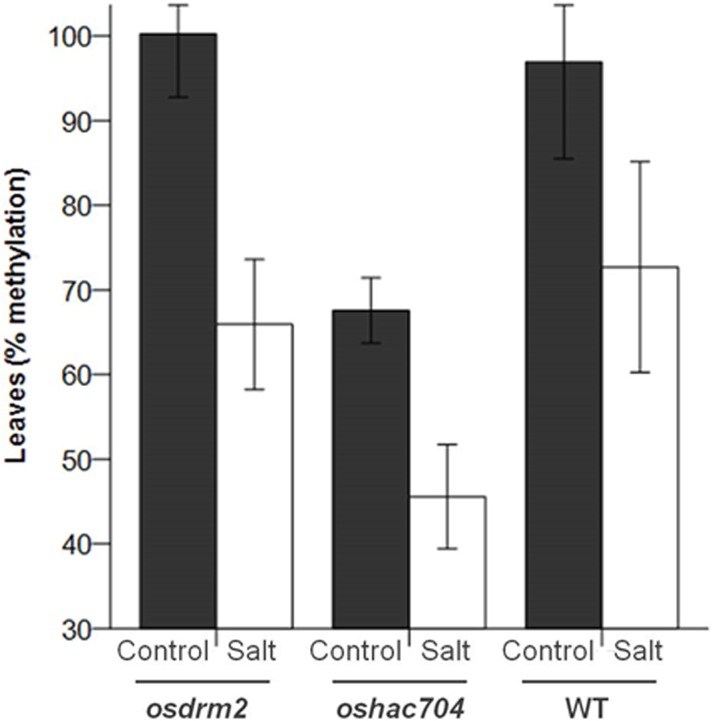
Global DNA methylation levels in rice mutants. Leaves of the *osdrm2* and *oshac704* rice mutants and WT (Dongjin) in control or salt stress conditions (24h of 200 mM NaCl) were used. The methylation values represented in the plots correspond to percent methylation of the samples relative to a methylated control DNA supplied with the kit. There were statistically significant differences between varieties (F(2, 18) = 6.628; p = 0.007) as well as treatment (F(1,8) = 14.307; p = 0.001). The control samples always had higher % of methylation than the salt stress ones. The *oshac704* mutant had the lowest methylation percentage (p<0.05).

Lower levels of DNA methylation were observed in the *oshac704* mutant in control conditions as compared to WT (p<0.05) (Fig 5). There were no significant interactions between mutants/WT and treatment (F(2,18) =. 285; p = 0.755) meaning that the treatment effect was not different for the mutants and WT and consisted on DNA demethylation under salt stress. For example, regarding the *hac704* mutant, it has undoubtedly less methylation than the WT, losing 32% of methylation with salt stress. The methylation in control is 67.6% (relative value) representing the absolute methylation that the mutant actually has. Under salt stress, the methylation is 45,6% (relative value) and thus, for *oshac704*, the loss of methylation under salt stress was about 32%. Interestingly, the salt- stress related demethylation registered in the mutants (34% and 32% for *osdrm2* and *oshac704*, respectively) was more extensive than in the WT (25%) (Fig 5).

**Fig 6:**
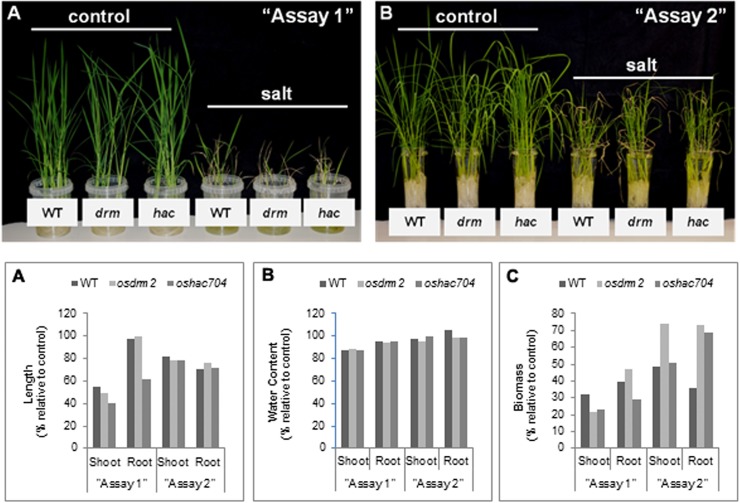
Phenotypic evaluation of epigenetic rice mutants under salt stress. The assays “1” and “2” (A and B, respectively) refer to the application of salt stress at distinct developmental stages for different time periods. The length and biomass values correspond to a % of change in salt relative to control. For details, see description in material and methods. (C) Shoot and root length of WT, *osdrm2* and *oshac704* in salt stress relative to control conditions. (D) Percentage of water content in shoots and roots of WT, *osdrm2* and *oshac704* in salt stress relative to control conditions. (E) Biomass of shoots and roots of WT, *osdrm2* and *oshac704* in salt stress relative to control conditions.

For the functional characterization of *osdrm2* and *oshac704* mutants, the salinity tolerance was evaluated at the seedling stage as described in the material and methods. Two salt stress assays, differing on the plant developmental stage at which salinization is imposed and duration of salt stress period, were performed as described in methods section. The modified standard evaluation score was used for rating the visual symptoms of salt toxicity (22). At the end of”Assay 1” all plants showed a complete growth cessation with most leaves drying, and thus were classified as susceptible. In comparison to mutants the WT showed slightly lower symptoms of salt injury (Table 1 and Fig 6A). In “Assay 2”, when salinization was imposed to 12 days-old seedlings, growth retardation was observed but most leaves maintained the green color with rolled tips. Compared to WT, the mutants showed a slightly better performance under stress conditions as long as the stress was imposed to 12 days-old seedlings (Table 1 and Fig 6B). A slightly enhanced performance of epi-mutants may well be linked to their higher flexibility in changing DNA methylation levels under stress.

Concerning the salt effects on growth, in both assays the mutants showed a marked reduction of shoot length when compared to WT. The root length was similarly affected by salinity in WT and mutants in “Assay 2”, but in “Assay 1” the *oshac704* mutant exhibited a severe growth reduction as compared to WT and *osdrm2* mutant (Fig 6C). The percentage of water content was not substantially different between WT and mutants in both assays (Fig 6D). In “Assay 1”, the mutants showed reduced biomass as compared to WT (except for roots of *osdrm2*), while in “Assay 2” the opposite results were observed (Fig 6E). Statistical analysis was performed by one-way ANOVA to calculate the effects of the treatment (control *versus* salt imposition) and the mutations [WT (Dongjin) *versus* mutants]. Statistical significance was assumed for p<0.05. The detailed statistical analysis of Fig 6C–6E is presented in S4 Table.

## Discussion

In this study, we evaluated global DNA methylation levels in distinct tissues of salt tolerant and sensitive rice varieties upon salt stress imposition. Global DNA methylation levels were quantified by the ELISA-based technique assay. This experimental approach has been widely used to measure DNA methylation in cancer research because it is relatively inexpensive and enables a fast, reliable and accurate processing of a high amount of samples [26]. The quantification of global DNA methylation levels by an ELISA assay is still quite new in plants and so far, it was only used to quantify DNA methylation of cork oak genome [27]. In the present work, shifts in global DNA methylation levels were detected after salt stress imposition. In addition, these shifts were influenced by genotype and tissue type. The salt tolerant rice variety Pokkali was able to rapidly reduce DNA methylation under salt stress while the salt-sensitive ‘IR29’ showed a low ability to adjust DNA methylation levels upon salt stress suggesting a link between the plasticity of DNA methylation and plant performance under salt stress. Previous studies in rice using the MSAP technique reported DNA demethylation events upon salt stress imposition [12–13]. Also, in rapeseed, salt-induced demethylation assessed by MSAP markers was much stronger in the tolerant variety Exagone as compared with the sensitive ‘Toccata’ [28]. Our cytological analyses revealed a spatial reorganization of DNA methylation patterns in response to salt stress which is consistent with the reorganization of heterochromatic domains in rice interphase nuclei after salt stress imposition or treatment with the 5-azacytidine (5-AC) hypomethylating drug [10].

DNA methylation levels were significantly lower in root tissues than in leaves. Tissue-dependent DNA methylation patterns have also been previously reported but a convincing explanation for those variations is still missing [11,13,29]. Some authors have argued that tissue-specific biological functions should imply a tissue-specific gene regulation, eventually involving differential DNA methylation [30]. Comparative transcription profiling under salinity stress showed that shoots and roots operate differently between rice varieties. For example, shoots of susceptible varieties have a higher number of salinity-induced transcripts than those of tolerant varieties [17], opposite to what was observed for roots of the same varieties (‘FL478’ and ‘IR29’) [31]. Therefore, a tissue-dependent DNA methylation pattern may not exclusively explain tissue-specific gene regulation. Another possible explanation for tissue-dependent DNA methylation pattern relates to tissue complexity and differentiation. The presence of undifferentiated meristematic tissue in roots could also explain the lower methylation levels observed in this tissue as compared to leaves, since meristematic root tip cells tend to have more open chromatin states and less DNA methylation [32].

Demethylation events occurred mainly on TE-related loci and these genetic elements comprise approximately 30% of the rice genome (http://rice.plantbiology.msu.edu/). More interestingly, a LTR located in the centromeric region of chromosome 12 (Chr12-centromere-like LTR) failed to be amplified in ‘Pokkali’ under salt stress conditions which may suggest a salt-induced transposition event. It is well known that stress can cause widespread genomic restructuring events including transposition of mobile elements [33] and in rice, mainly *in vitro* cell or tissue culture have been associated with transposition [34–37]. In addition, this LTR was undetectable in the IR29 variety, which could be related to modified transposition sites. Different transposition sites between varieties have been reported in other plant species, namely in Arabidopsis, where significant transposition events were detected between ‘Col’ and ‘Ler’ ecotypes [38].

It is not clear whether changes in DNA methylation are simple indirect effects of salt stress or a mechanism for regulation the expression of salt stress responsive genes. The production of reactive oxygen species (ROS) has been associated with stress [39–41] and may affect DNA methylation levels [42]. During carcinogenesis, the 8-hydroxyguanosine, a product of oxygen radical damage, is able to replace guanine leading to altered DNA methylation patterns [43,44]. However, because DNA demethylation occurs as early as 1h after salt stress independently of DNA replication, indicates that chromatin remodeling enzymes should be involved, specifically the ones involved in active demethylation [45]. In fact, our results point to an active demethylation in the tolerant ‘Pokkali’, since we observed an induction of DNA demethylases by salt stress imposition. In contrast, the susceptible ‘IR29’ responded to salt stress with the transcriptional induction of both DNA demethylases and DNA methyltransferases, which could explain the non-significant alteration of global DNA methylation levels.

Rice plants with mutations for epigenetic modulators are ideal tools to investigate putative links between epigenetic marks and effects at phenotypic traits. The rice mutant line 3A-08043 has a silenced *OsDRM2* gene, an MTase of the Domains Rearranged Methyltransferases (DRM) family involved in *de novo* methylation in all sequence contexts [46–49]. Due to the *OsDRM2* gene silencing, it would be expected a reduction of global DNA methylation levels in this line when compared to WT. However, in this study, we detected similar methylation levels between the mutant and the WT which could be explained by the presence of other members of this MTase family in rice [50]. Likewise, it was previously shown that knockout or knockdown of *OsDNG701*, a rice DNA glycosylase responsible for DNA demethylation, did not cause global DNA hypermethylation but rather a locus-specific DNA hypermethylation, namely in Tos17 [24]. Also in Arabidopsis, mutations in the DNA demethylation pathway affected only specific genes [51–54]. Together with our results, it may well be possible that the loss of function of some chromatin remodelling enzymes can affect the writing and/or erasing of epigenetic marks in a locus-specific manner instead of genome wide effects. Interestingly, the rice mutant for the histone acetyltransferase revealed significantly lower global methylation than WT, illustrating the crosstalk between distinct epigenetic marks.

Regarding the phenotype of mutant plants, despite the marked reduction in spikelet fertility and impaired seed germination and development (particularly evident in the *osdrm2* mutant) plants were able to generate progeny. Contrary to mammals, where mutations in methyltransferases have lethal effects [55], in plants and fungi mutations in single DNA methyltransferases do not cause significant impacts in phenotype [56–58]. However, when looking at specific phenotypic parameters, it was possible to detect that root length and biomass of the *osdrm2* mutant was less affected by salinity than the WT.

In summary, the tolerant rice variety Pokkali exhibited a higher capacity for changing DNA methylation levels in response to salt stress. In contrast, the salt sensitive IR29 variety was unable to adjust its methylation levels. These findings suggest different epigenetic regulatory networks between rice varieties and may account for the variability of salt stress response/tolerance observed in rice. Further studies are needed to better understand the regulation of epigenetic marks and their impact in salt stress adaptation.

## Supporting Information

S1 Fig Schematic representation of the rice T-DNA insertion lines (http://signal.salk.edu/cgi-bin/RiceGE). (EPS)

S2 Fig Expression studies of *OsDRM2* and *OsHAC704* in the T-DNA rice mutant lines. (EPS)

S3 Fig Spikelet fertility in WT (Dongjin) and T-DNA rice mutant lines. (EPS)

S4 Fig Threshold cycle (CTs) values for the ubiquitin-conjugating enzyme E2 (UBC2) and elongation factor (eEF) genes under salt stress conditions. (EPS)

S1 Table List of Primers used for expression studies of DNA demethylases and DNA methyltransferase. (DOCX)

S2 Table List of Primers used in the McrBC methylation analysis. (DOCX)

S3 Table List of Primers used for genotyping rice T-DNA insertion lines. (DOCX)

S4 Table Statistical analysis underlying phenotypic evaluation of rice mutants. (DOCX)
