# Supplementary material for: Salt Tolerant and Sensitive Rice Varieties Display Differential Methylome Flexibility under Salt Stress
Source: PLoS One. 2015 May 1;10(5):e0124060. doi: 10.1371/journal.pone.0124060 (PMC4416925; doi:10.1371/journal.pone.0124060)
Supplement: S1 Table — (DOCX) [file pone.0124060.s005.docx]

S1 Table

| **Genes** | **Primer forward** | **Primer reverse** | **References** |
| --- | --- | --- | --- |
| DNG701 | ATGGCGAAAGACGAGAACCC | TCGCACTGACCATGGGAAAG | This work |
| DNG710 | CCTGCACTGACTGAACATGG | CTGGGAGCATCTGAAGAGGA | This work |
| OsDRM2 | GTGGGCCATCTGGGAATCAA | CCTCTCATTGGGCAAGCTGA | This work |
| OsUBC2 | TTGCATTCTCTATTCCTGAGCA | CAGGCAAATCTCACCTGTCTT | This work |
