# Supplementary material for: Salt Tolerant and Sensitive Rice Varieties Display Differential Methylome Flexibility under Salt Stress
Source: PLoS One. 2015 May 1;10(5):e0124060. doi: 10.1371/journal.pone.0124060 (PMC4416925; doi:10.1371/journal.pone.0124060)
Supplement: S2 Table — (DOCX) [file pone.0124060.s006.docx]

**S2 Table**

| **TEs / repetitive sequences** | **Primer forward** | **Primer reverse** | **References** |
| --- | --- | --- | --- |
| TE-I_Os04g19320 | GCCAGCTCCTCTCTCTCTCA | AGATACGGCTATTGGCGATG | [1] |
| TE-I_Os04g17620 | AATCCTGAGCTTCCAGCAGA | TCAGGTGGAGTTTTGTGCAT | [1] |
| TE-II_Os04g08710 | CCATCCTCTTACAGGGACCA | CCCGTCTTCCTCATTGATCT | [1] |
| Chr3-AnacA2_TE | TCTTCCTCCTCCCTCTCCTC | CTCGCGACTTCGATTAGACC | This work |
| Chr8-Tnr8_TE | TTGCAAAACAACAAAAATCTTGA | ACATTATTTCTGTAAATGGTGTATG | This work |
| Chr9-Ty3-gypsy_TE | TGTTATTTGYATTGATTTAATAGGG | ACTACCTCCCTCTCTCTCTCA | This work |
| Chr12-centromere-like_LTR | GTCACTCCCCGACCCTATCT | GCCGAGCTTCTTCTTGTCAC | This work |
| Tos17 | GTCCCTATCCATGTGCTGGT | TGTTTACGCTCAGCAACACC | This work |
| Telomere_rep.seq. | CCCCAACCTTAATGAAGCAA | CATCATAGCCCCCGTTGTTA | This work |
| **Genes** |  |  |  |
| *OsRMC* | GAAGACCTGGTTCCGATTGA | CTGATGTTGGTGCTGCTCAT | This work |
| *OsHKT1,5* | TCCGACGTCCTAACCCTAGCTCTAC | TGGTGATGATTTACGCACATGAGAC | [2] |
| *OsSalT* | TTCCAGACCTTCCAAAGAATCCAA | ACAAGGAAATTTAAGCGACCACGA | [2] |
| *OsNHX1* | GCGGATCCACCTGGACTATC | GAAATCGGGATTTGGTATCGAG | [2] |
| *eEF* | ACCCTCCTCTTGGTCGTTTT | AAATACCCGCATTCCACAAC | [3] |
| *OsActin* | CCTCAGCCGCCTTTCACTAT | CATCTGTGGTGATGTGGCGA | This work |

1. Yin BL, Guo L, Zhang DF, Terzaghi W, Wang XF, Liu TT, et al. Integration of cytological features with molecular and epigenetic properties of rice chromosome 4. *Mol Plant* 2008; 1(5): 816-829.

2. Negrão S, Courtois B, Ahmadi N, Abreu I, Saibo N, Oliveira MM. Recent updates on salinity stress in rice: from physiological to molecular responses. Crit Rev Plant Sci 2011; 30: 329–377.

3. Figueiredo DD, Barros PM, Cordeiro AM, Serra TS, Lourenço T, Chander S et al. Seven zinc-finger transcription factors are novel regulators of the stress responsive gene OsDREB1B. *J Exp Bot* 2012; 63(10): 3643-3656.
