# Supplementary material for: Salt Tolerant and Sensitive Rice Varieties Display Differential Methylome Flexibility under Salt Stress
Source: PLoS One. 2015 May 1;10(5):e0124060. doi: 10.1371/journal.pone.0124060 (PMC4416925; doi:10.1371/journal.pone.0124060)
Supplement: S3 Table — (DOCX) [file pone.0124060.s007.docx]

S3 Table

| **T-DNA line** | **Genes** | **Primer forward** | **Primer reverse** | **References** |
| --- | --- | --- | --- | --- |
| 3A-08043 | OsDRM2 | ACGCTTCAGTGGGTAACTGC | CTGCTGACATGGTAGCCTTT | This work |
| 4A-01884 | OsHAC704 | GGCTCAACATCAGCAACGTC | TCCGATACACCATACGGGGA | This work |
