# Supplementary material for: Salt Tolerant and Sensitive Rice Varieties Display Differential Methylome Flexibility under Salt Stress
Source: PLoS One. 2015 May 1;10(5):e0124060. doi: 10.1371/journal.pone.0124060 (PMC4416925; doi:10.1371/journal.pone.0124060)
Supplement: S4 Table — (DOCX) [file pone.0124060.s008.docx]

**S4 Table**.

|  | **Effects of the treatment**  Control *versus* Salt | | **Effects of the mutations**  WT (Dongjin) *versus* Mutants | |
| --- | --- | --- | --- | --- |
|  | **“Assay 1”** | **“Assay 2”** | **“Assay 1”** | **“Assay 2”** |
| **Shoot length**  **(cm)** | F(1,52)=570.847  p<0.001 | (F(1,104)=155.598 p<0.001 | F(2,52)=5.717 p=0.006 | F(2,104)=7.599 p<0.001 |
| **Root length**  **(cm)** | F(1,52)=21.68  p<0.001 | F(1,52)=175.212 p<0.001 | F(2,52)=22.669 p<0.001 | F(2,52)=5.749 p<0.001 |
| **Shoot water content**  **(%)** | (F(1,52)=154.317 p<0.001 | F(1,104)=36.495 p<0.001 | F(2,52)=4.775 p=0.012 | F(2,104)=9.799 p<0.001 |
| **Root water content**  **(%)** | F(1,52)=46.728  p<0.001 | F(1,104)=0.907 p=0.343 | F(2,52)=5.49 p=0.007 | F(2,104)=5.59 p=0.005 |
| **Shoot biomass**  **(g)** | F(1, 52)=189.246  p<0.005 | F(1,104)=303.536 p<0.001 | p>0.05 | F(2,104)=29.338 p<0.001 |
| **Root biomass**  **(g)** | F(1,52)=94.616  p<0.001 | F(1,104)=22.76 p<0.001 | F(2,52)=5.658  p<0.001 | F(2,104)=13.656 p<0.001 |

Statistical analysis by one-way ANOVA to calculate the effects of the treatment (control *versus* salt imposition) and the mutations (WT (Dongjin) *versus* mutants). Statistically significance was assumed for p<0.05.
